# Sexual Neurasthenia

**Published:** 1895-03

**Authors:** 


SEXUAL NEURASTHENIA (NERVOUS EXHAUSTION): ITS HYGIENE, CAUSES, SYMPTOMS, AND TREATMENT. With a chapter on Diet for the Nervous. By George M. Beard, A. M., M. D. Edited with notes and additions by A. D. Rockwell, A. M., M. D. Fourth Edition, with formulas. New York: E. B. Treat, 5 Cooper Union. 1895. Price, $2.75.

In the November number of this journal, at page 371, was given a review of the famous work on neurasthenia by George M. Beard, M. D. Now we present to the notice of our readers its companion work, *Sexual Neurasthenia*. This work is sometimes confounded with the other one, but it is not the same, being a treatise upon a specialized form of neurasthenia. As said in the preface, "few morbid conditions cause more unhappiness, or are attended with more disastrous results than disturbances of the sexual function. He who sees only here and there, and now and then, cases of this kind has but little conception of the vast sum of misery around him which is either silently borne, or finds expression in some mad act of crime or in suicide. I do not so much refer to those cases of sexual perversion which assume such protean forms, and which have been so graphically described by Kraft-Ebing. These are mostly cases of voluntary self-abasement. [See "Review of Psychopathia Sexualis," by Kraft-Ebing, in THE HOMŒOPATHIC PHYSICIAN for September, 1893, page 478.] * * * There are, on the contrary, a large number of unfortunates who, without fault or wish of their own, are in a continual state of sexual erethism that is abnormal and pathological."

From this quotation a view will be obtained of the motive of the book and of its value.

The third edition, it may be well to state, was reviewed in this journal in September, 1891, page 374.

